# Study on Nature-inspired Fractal Design-based Flexible Counter Electrodes for Dye-Sensitized Solar Cells Fabricated using Additive Manufacturing

**DOI:** 10.1038/s41598-018-35388-2

**Published:** 2018-11-19

**Authors:** Sagil James, Rinkesh Contractor

**Affiliations:** Department of Mechanical Engineering, California State University Fullerton, Fullerton, CA 92831 USA

## Abstract

Dye-Sensitized Solar Cells (DSSC) are third generation solar cells used as an alternative to traditional silicon solar cells. DSSCs are characterized by their durability, easy handling and ability to perform better under diverse lighting conditions which makes them an ideal choice for indoor applications. However, DSSCs suffer from several limitations including low efficiencies, susceptibility to electrolyte leakage under extreme weather conditions, and the need for expensive materials and fabrication techniques which limits their large-scale industrial applications. Addressing these limitations through efficient design and manufacturing techniques are critical in ensuring that the DSSCs transform from the current small-scale laboratory levels to sizeable industrial production. This research attempts to address some of these significant limitations by introducing the concepts of nature-inspired fractal-based design followed by the additive manufacturing process to fabricate cost-effective, flexible counter electrodes for DSSCs. The new conceptual fractal-based design counter electrodes overcome the limitations of conventional planar designs by significantly increasing the number of active reaction sites which enhances the catalytic activity thereby improving the performance. The fabrication of these innovative fractal designs is realized through cost-effective manufacturing techniques including additive manufacturing and selective electrochemical co-deposition processes. The results of the study suggest that the fractal-based counter electrodes perform better than conventional designs. Additionally, the fractal designs and additive manufacturing technology help in addressing the problems of electrolyte leakage, cost of fabrication, and scalability of DSSCs.

## Introduction

Solar cells are leading innovation to renewable energy considering the recent advances in improving efficiencies of the solar panels along with the drastic decrease in the cost^1^. These efforts are crucial to the future of energy production because it could minimize the dependence on fossil fuels and other types of non-renewable resources^1^. Currently, solar cells are being used in different sectors of industries such as automotive, military, outdoor advertising, electronics, apparel designing and several other applications^2^. Most commercial solar cells are first generation solar cells made of crystalline silicon (c-Si)^2^. The solar panels made of c-Si cells are considerably expensive regarding initial cost^3^. Moreover, the c-Si cells are rigid, fragile and have low absorption coefficients. Thin film solar cells, referred to as second-generation solar cells, overcome some of these limitations. However, thin film solar cells still suffer from drawbacks including high costs of active materials, scarcity of critical elements and poor performance under cloudy weather^4^. Third generation solar cells are an attractive replacement for both first and second generation solar cells. These solar cells use a wide variety of materials including polymer materials, conductive plastics and organic dyes^5^. They are cost-effective and are the most suitable solar cells for low-density applications such as building-integrated applications, rooftop collectors, commercial indoor electronic products, portable devices, and so on^4^. Among various types of third generation solar cells, Dye-Sensitized Solar Cells (DSSC) have gained much attention recently^6^. DSSCs have low manufacturing and material cost and are environment-friendly^6^. They are flexible and easier to handle and less susceptible to damage and perform better under a wide range of lighting conditions^6^.

**Figure 1:**
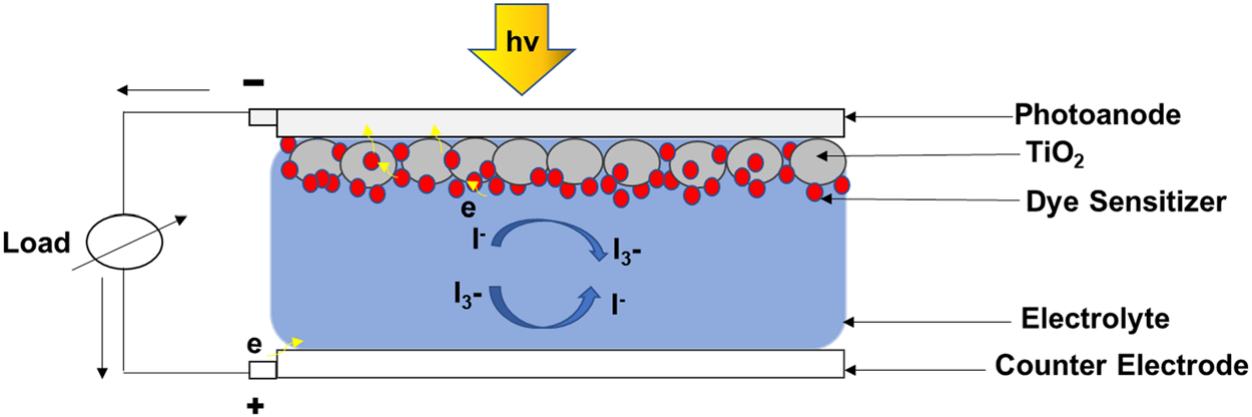
Schematic of Operational Principle of Dye-Sensitized Solar Cell.

Unlike silicon solar cells, DSSCs uses the method of artificial photosynthesis to capture the solar energy^7^. DSSCs primarily consists of a Top Electrode (Photoanode), Counter Electrode (CE), porous Titanium dioxide (TiO_2_) layer, dye pigments, a liquid electrolyte, and catalyst material^6^. Energy conversion in DSSCs happens when the incident light is absorbed by the dye pigments coated on the surface of porous TiO_2_ particles. Photoexcitation of dye leads to electron transport into the conduction band of TiO_2_. An electrolyte gel solution (iodine/tri-iodide redox couple) is used to reduce the dye cation (regeneration of the ground state of dye). Iodide ions, which oxidize in this reaction, are regenerated at the CE. The CE collect electrons from the external circuit and catalyze the reduction of tri-iodide ions to iodide ions. The schematic of the operational principle DSSC is shown in Fig. 1.

DSSC can be manufactured in small quantities using a combination of techniques including roll-to-roll processing, doctor blading, spin coating, ink-jet printing and screen printing technologies^8^. However, scaling-up the DSSC manufacturing from small-scale laboratory tests to sizeable industrial production requires better and efficient manufacturing processes. Moreover, the efficiency of DSSC is less (10–12%) compared to commercial silicon cells (18–20%)^4^. Other limitations of DSSCs include short lifetime, susceptibility to fracture (in the case of glass-based electrodes), use of liquid electrolyte medium resulting in leakage, occasional freezing during cold weather, and thermal expansion during hot weather conditions^9^. Additionally, most DSSC electrodes are made using Indium-Tin-Oxide (ITO) film or Fluorine-doped Tin Oxide (FTO) film^10^. These materials are limited by their brittleness, relatively expensive to manufacture, need for high-temperature treatments, toxic (as in the case of FTO), and poor conductivity^11^. Overall, the performance of DSSCs are affected by several critical factors including a reduction in recombination loss in photoanodes, absorptivity, stability, and electrocatalytic activity of CEs^12^.

Among the components of DSSCs, CEs play a critical role in electrons/ions transportation and electron injection at TiO_2_/dye interface. The functions of a CE in DSSC include i) acting as a positive electrode for collecting electrons, ii) providing catalysts for enhancing charge transfer rate and iii) acting as a reflector for unabsorbed light^12^. Consequently, the optimal CEs for DSSCs should possess qualities such as high catalytic activity, high electrical conductivity, large surface area, optimal thickness, excellent stability and low fabrication cost^12^. The DSSC CEs typically consist of a conductive substrate layer on which the catalyst material is deposited. Most CE substrates are made of glass coated with a thin layer of ITO or FTO material^12^. Recently, other materials including metals such as carbon steel, stainless steel, copper (Cu) foils have also been used for CEs with admirable performance^13^. The CE catalysts help in reducing the tri-iodide ions to iodide ions at the CE/electrolyte interface. Conventional DSSC CEs use Platinum (Pt) are the catalyst material owing to its superior catalytic activity and high electrical conductivity^14^. However, Pt material is prohibitively expensive thereby limiting the possibilities of extensive use in DSSCs.

Currently, there is massive research interest in replacing Pt with other low-cost materials along with finding alternative strategies to enhance their catalytic activity^14,15^. Some of the low-cost Pt-free catalyst materials include carbon materials – graphite, carbon black, conductive polymers – Polypyrrole (PPy), poly(3,4-ethylene dioxythiophene) (PEDOT), transition metal compounds (TMCs) and their hybrids^14,15^. It was reported that the PPy based CE showed better catalytic properties and less charge transfer resistance compared to a Pt-based CE^16^. Graphite has the advantage of being low-cost material, and studies have reported that using graphite as the catalyst material helps in enhancing the electronic conduction and energy conversion efficiency of DSSCs^17^. DSSC CEs fabricated based on TMC catalyst materials such as oxides, nitrides, sulfides, and carbides have exhibited better performance compared to their Pt-based counterparts^14^.

The catalytic activity of CEs can be further enhanced by increasing their surface area^15^. The number of catalytically active sites would significantly increase for larger surface area resulting in more reactions to reduce the tri-iodides the CE/electrolyte interface. For a given amount of catalyst, their catalytic activity can be enhanced by maximizing the density of active sites on the CE surface. Studies done on catalyst distribution has reported that the catalytic activity increases up to a certain size (typically in the range of nanometers) and then rapidly decline as the size of the catalyst structure increases^18^. Smaller catalyst size would expose more active sites without increases in the volume of the material. A study done on DSSCs using a three-dimensional (3D) conductive grid design based CE showed higher catalytic activity, faster charge transfer rate, and higher energy conversion efficiency compared to a conventional planar CE based DSSC^19^. The study used Pt nanoparticles as the catalyst material and found that the 3D grid design provided larger active sites for the catalytic reaction. Another study used a 3D spiral-shaped design using Titanium (Ti) wire for the CEs and found superior light-harvesting performance for the DSSCs^20^.

Deposition of catalyst materials on CEs in DSSCs is primarily done using techniques such as Spin Coating, Chemical Vapor Deposition (CVD), Physical Vapor Deposition (PVD), *in-situ* polymerization and so on^12^. However, these processes have several limitations including difficulty in creating complex structures, improper deposition, material loss, poor surface adhesion, and production of hazardous by-products^11^. Moreover, these techniques need to be supplemented by additional processes such as masking, etching, and so on to achieve proper deposition^12^. Most importantly, all these processes can be very expensive and cannot be done without high-end vacuum equipment. Nevertheless, there is an increased need for simple strategies involving efficient CE designs along with low-cost manufacturing solutions for the widespread commercialization of DSSC technology.

In recent years, there has been increasing interest in integrating nature-inspired fractal designs into engineering applications including optoelectronics, stretchable electronics, antenna design, architecture designs, tissue engineering, heat-transfer channels and so on^21–23^. Fractals are rough structures extensively found in nature including the leaf venations, and they represent self-similarity in which each sub-structure resembles the whole. The fractal design for engineering structures provide better stability, optimal surface coverage, uniform ion transportation, and facilitate the efficient collection of thermal and electrical energy^24^. Incorporating the fractal design into CEs in DSSC would potentially maximize the active surface to volume ratio, reduce electrolyte ionic path, and enable faster electron injection resulting in better performance. Application of bio-inspired fractal electrodes for thin film amorphous silicon solar cells have been demonstrated in the past and the results suggest that the attained energy density is significantly higher (30 times) compared to conventional planar electrodes^25^. Another study used quasi-fractal structure based electrodes and suggested that increasing the number of hierarchical orders of fractal would help reduce the electrical resistance significantly^11^. Quasi-fractal designs have also been used for fabricating metallic networks for transparent electrodes for flexible electronics and photovoltaic applications^26^. These studies suggested that the use of fractal designs would impart flexibility, provide mechanical strength, and increase the electro-optical performance of the electrodes.

Fabrication of structures based on fractal designs is extremely challenging and quite cumbersome^27^. Past efforts on fabricating fractal structures involved multiple processes including chemical etching and metal film deposition through sputtering followed by transferring the pattern onto the substrate using the drum printing technique^24^. Employing these process for fabricating fractal structures is certainly expensive, needs vacuum processing and thus limits the scalability of the technology^26^. Additive Manufacturing (AM) also referred to as 3D Printing, on the other hand, has been playing a pivotal role in recent manufacturing advancements. 3D printing technologies have become very popular in the past few years and are now being used in several applications including electronics, architecture, and biomedical and so on^28^. These 3D printing techniques can overcome the limitations of conventional fabrication techniques and provides the limitless potential for creativity at relatively low cost while minimizing material usage. The capabilities of AM technologies can be extended for the creation of complex shapes including fractals. Recent studies have reported the use of 3D printing technique to fabricate fractal designs generated through Iterated Function System (IFS)^29^. Another study used Selective Laser Melting (SLM) based 3D printing technique was used to fabricate 3D fractal antennas and suggested that AM technologies reduce material consumption and cost while fabricating fractal structures^30^. Hence, 3D printing technologies open possibilities of fabricating customized fractal design based structures while reducing the cost of manufacturing.

The goal of this research is to address some of the existing limitations of DSSC technology including low-efficiency, high fabrication cost, electrolyte leakage, and need for expensive or toxic raw materials. The specific objectives of this research are 1) to study the feasibility of incorporating nature-inspired fractal based designs for the Counter Electrodes (CEs) used in DSSCs and 2) to demonstrate the applications of low-cost manufacturing techniques such as Additive Manufacturing (AM) and Selective Electrochemical Co-deposition process for fabricating flexible CEs for DSSCs. The proposed leaf-mimicking fractal-based design aims to enhance the catalytic activity on the CE and improve the performance and efficiency of the solar cells. Different geometrical designs including two-dimensional (2D) and three-dimensional (3D) fractal-designs are studied to understand the performance of the CEs. The fabrication of the CEs is done using Fused Deposition Modeling (FDM) based AM technique followed by a selective electrochemical co-deposition process. The fabricated CEs are characterized, evaluated and then integrated into the DSSC assembly for performance testing.

## Research Methodology

In this study, the DSSC CEs are designed based on the nature-inspired fractal design to improve its catalytic activity and overall performance. Four designs of CEs are used for this study including planar design, cross design, two-dimensional (2D) fractal design and 3D fractal design. The fabrication of the CEs is done using the AM technique. It is followed by a selective electrochemical deposition process where a thin layer of metal along with catalyst particles are deposited on the 3D printed structure. The fabricated CEs are characterized using a digital/optical microscope and a Scanning Electron Microscope (SEM). The electrical properties are measured and compared for all the four designs of CEs. Further, DSSC assemblies are completed using the fabricated CEs along with the TiO_2_ layer, dye pigments, and the photoanode. The photovoltaic performance of each DSSCs is then studied to understand the Current-Voltage (I-V) curves and the photoelectric conversion efficiencies.

### Designing of CEs for DSSC

**Figure 2:**
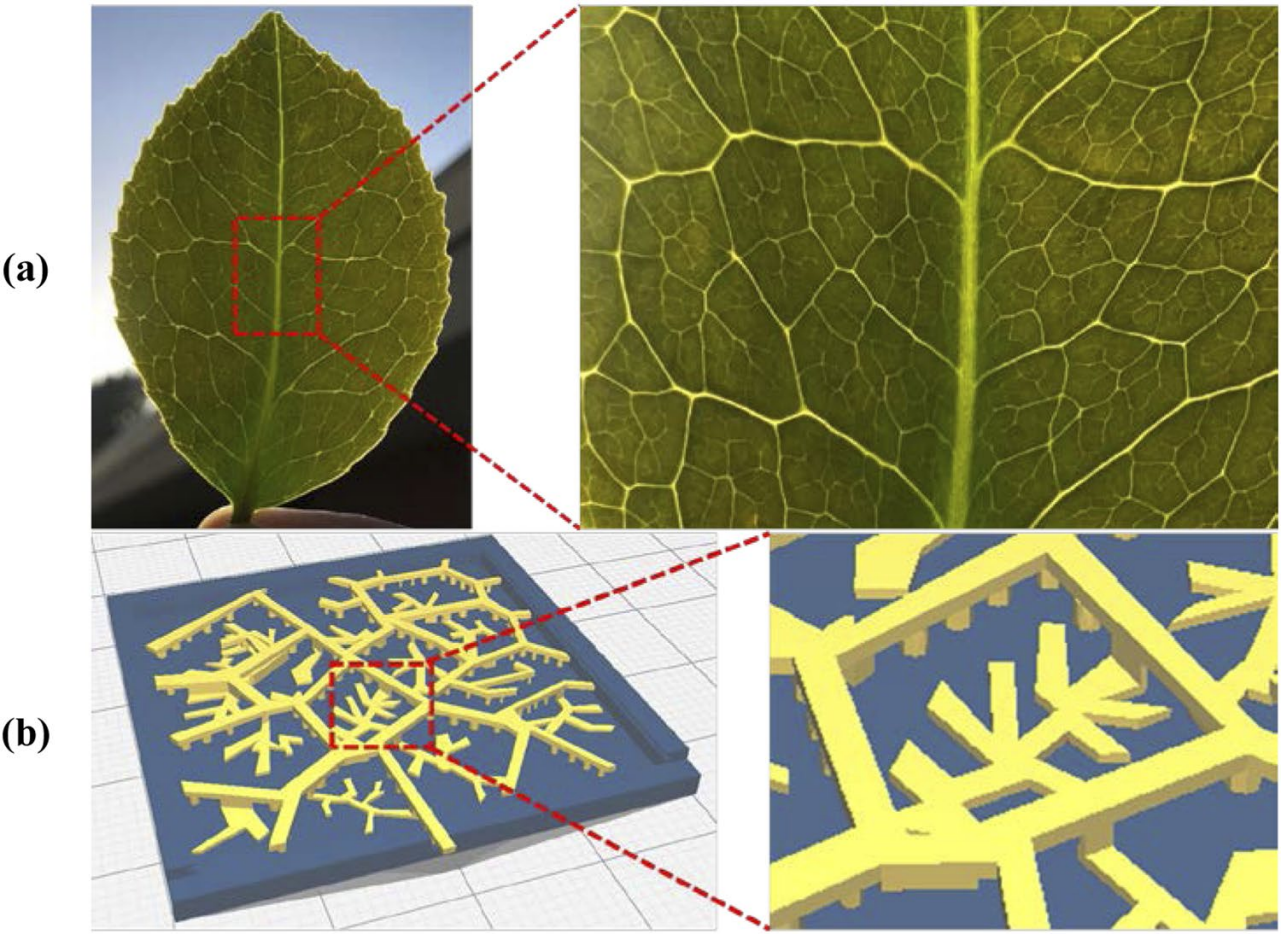
(**a**) Optical Images of *Camellia japonica* Leaf (inset: magnified view showing venation pattern) and (**b**) CAD Model of 3D Fractal Design for DSSC CE (inset: magnified view showing the sub-branches).

**Figure 3:**
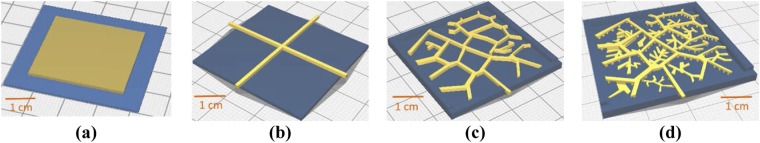
CAD model of DSSC CE designs (**a**) Planar (**b**) Cross (**c**) 2D Fractal and (**d**) 3D Fractal.

The fractal designs used for this study are inspired by the venation pattern found on the leaves of *Camellia japonica* (Japanese Camellia) plant. The Camellia plants have green oval, serrated leaves with an average length of 5 to 10 centimeters. The vein network on the leaves follows a pinnate venation having one primary vein extending from the base to the tip along with several prominent secondary veins branching to either side. Figure 2a shows the optical image of the *Camellia japonica* plant leaf. Past studies have reported that fractal parameters can be used to effectively characterize and describe the vein networks found on the leaves of *Camellia japonica*^31^. The motivation behind using the fractal design found on the leaves of *Camellia japonica* for the DSSC CEs is the potential ability of the structure to allow uniform ion distribution, provide optimal surface coverage and enhance the mechanical stability. Based on the venation pattern on the leaves of *Camellia japonica*, two fractal designs – 2D and 3D are considered for this study. While the 2D design provides structural stability for the CEs, the 3D design significantly increases the surface area along with maximizing the number of active catalytic sites on the CE surface as shown in Fig. 2b. It would facilitate the reduction of the tri-iodide ions of electrolyte resulting in a higher current generation. Moreover, the catalyst particles can be highly dispersed over a larger area within the 3D fractal structure which is beneficial when using expensive catalyst materials such as Pt. The fractal design for this study is modeled using the commercial software package – CATIA V5. The performance of the fractal design based CEs are compared with two other conventionally designed CEs – planar design and cross design. Figure 3 shows the CAD models of the four designs of CEs used in this study.

### Fabrication of CEs for DSSC

**Figure 4:**
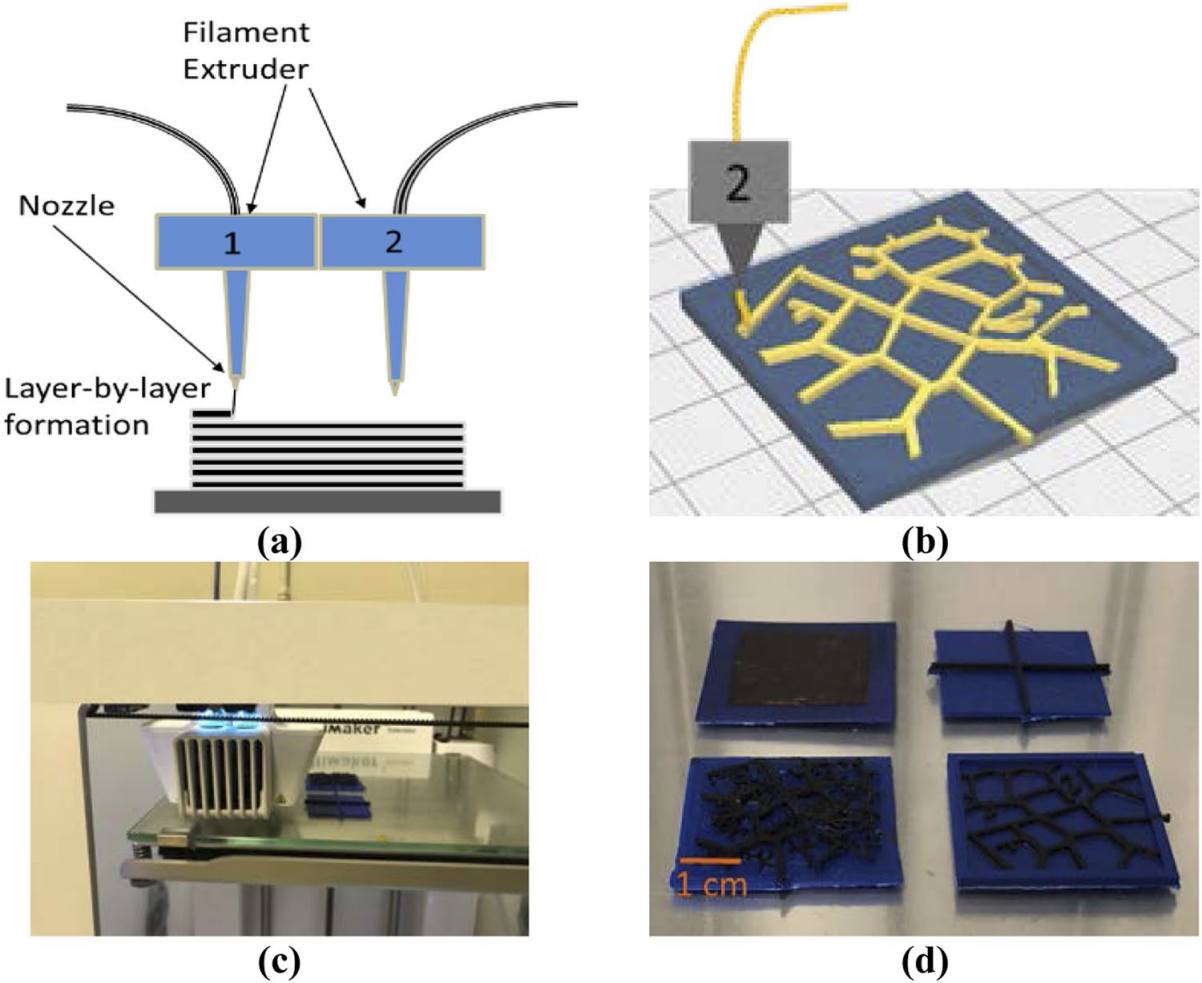
(**a**) Schematic of FDM 3D Printing Process (Double Extruder) (**b**) Schematic of 2D Fractal Structure Printed using Extruder 2 (**c**) FDM 3D Printing Process and (**d**) CEs Fabricated using FDM.

The CE for DSSCs is fabricated using AM technique followed by a Selective Electrochemical Co-deposition process. The 3D printing process used for this study is FDM which is the most suitable technique for printing polymer materials with high accuracy and at low cost. The FDM 3D printer used is Ultimaker 3 Extended with Dual Extrusion (make: Ultimaker^TM^, Netherlands). The designs of CEs are exported as.stl files and then converted to.gcode files using the software – Ultimaker Cura 3.0. The first extruder (extruder 1) is used for printing a base substrate made of non-conductive Acrylonitrile Butadiene Styrene (ABS) plastic filament. The second extruder (extruder 2) prints the CE structures using conductive black Polylactic Acid (PLA) filaments. The printing is done on a heated bed with temperatures of 80 °C and 70 °C for non-conductive ABS and conductive PLA filaments respectively. The layer thickness is set at 0.1 mm and extrusion temperature are maintained at 230 °C and 210 °C for non-conductive ABS and Conductive PLA respectively. The schematic of the 3D printing process used to print CEs for the DSSCs is shown in Fig. 4. The experimental conditions used for 3D printing the CEs through the FDM process are shown in Table 1.

**Table 1:** Process Parameters used for FDM 3D Printing of DSSC CEs.

Parameters	ABS	Conductive PLA
Bed Temperature, °C	80	70
Extruder Temperature, °C	230	210
Infill density, %	80	80
Layer height, mm	0.06	0.06
Build plate adhesion	Yes	No
Print speed, mm/sec	40	20

**Figure 5:**
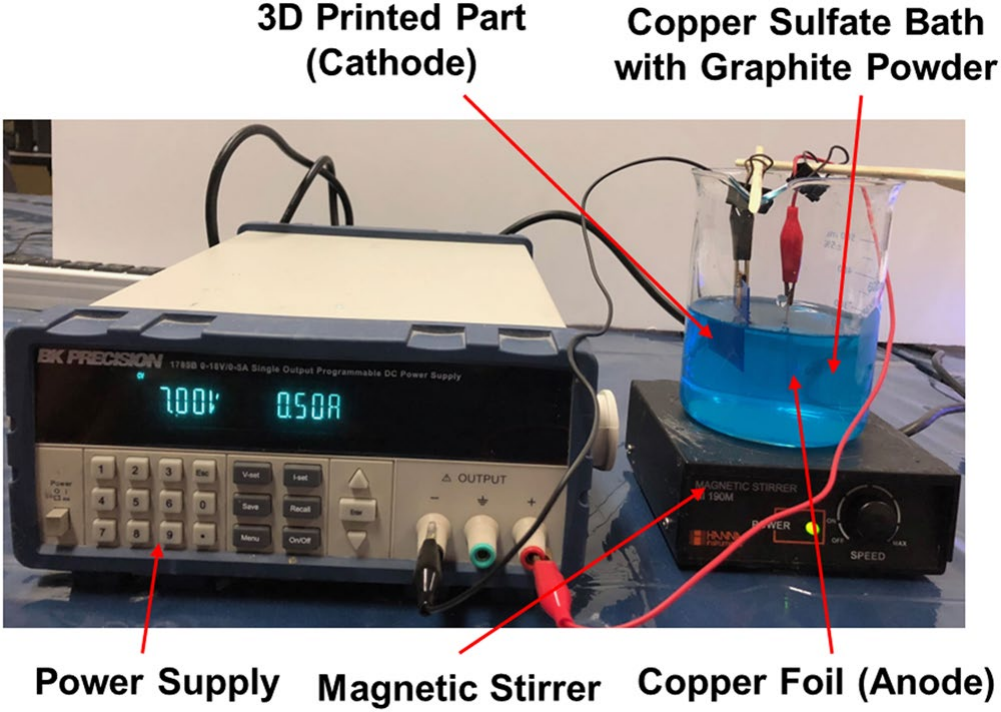
Experimental Set-up used for Selective Electrochemical Co-deposition Process.

The 3D printed CE need to be coated with a conductive material along with the catalyst particles. It would enhance the conductivity and the catalytic activity of the CEs during its operation. Selective electrochemical co-deposition is an electroplating process in which metal particles along with secondary phase particles are selectively coated over the target surface. This process is extremely cost-effective, produce uniform coatings efficiently without any residual stresses and without using toxic chemicals^32^. Moreover, the CEs in DSSC should have low interfacial resistance and strong adhesion of catalyst materials with the substrate. Therefore, selective electrochemical co-deposition is a suitable technique for the CE preparation. This technique is used in the current study to coat Cu/Graphite particles on the CEs fabricated through 3D printing. The choice of Cu and graphite materials are attributed to their high conductivity, low-cost, abundant supply, and long-term stability. During the experiment, a copper sulfate bath containing 250 ml of distilled water mixed with 50 grams of copper sulfate is stirred for 15–20 minutes. A Cu sheet is used as the anode while the 3D printed CE is the cathode. A DC programmable power supply (make: B&K Precision Corporation, USA) is used to apply a constant voltage during the deposition process. The experimental setup used for the selective electrochemical co-deposition process is shown in Fig. 5. The experimental conditions used for the selective electrochemical co-deposition process are shown in Table 2. After the initial deposition of a thin layer of copper on the cathode, 2 grams of micron-sized graphite particles (size 40–50 µm) are introduced into the copper sulfate bath while stirring constantly using a magnetic stirrer. A uniform thin layer of Cu/Graphite deposition is achieved by maintaining a constant distance (3 cm) between the anode and the cathode. The conductivities of the CEs are measured during the electrochemical co-deposition process. Morphology of the deposited Cu/Graphite on the CEs is examined using a Scanning Electron Microscope (SEM).

**Table 2:** Experimental Conditions Used for Selective Electrochemical Co-Deposition Process.

Parameter	Value
Anode	Copper Foil
Cathode	3D Printed CE
Electrolyte solution	Copper Sulfate
Second phase particles	Graphite Powder (40–50 µm)
Power supply (Voltage)	6–8 V
Inter-electrode gap	2–3 cm

## Results of Fabrication of CEs

**Figure 6:**
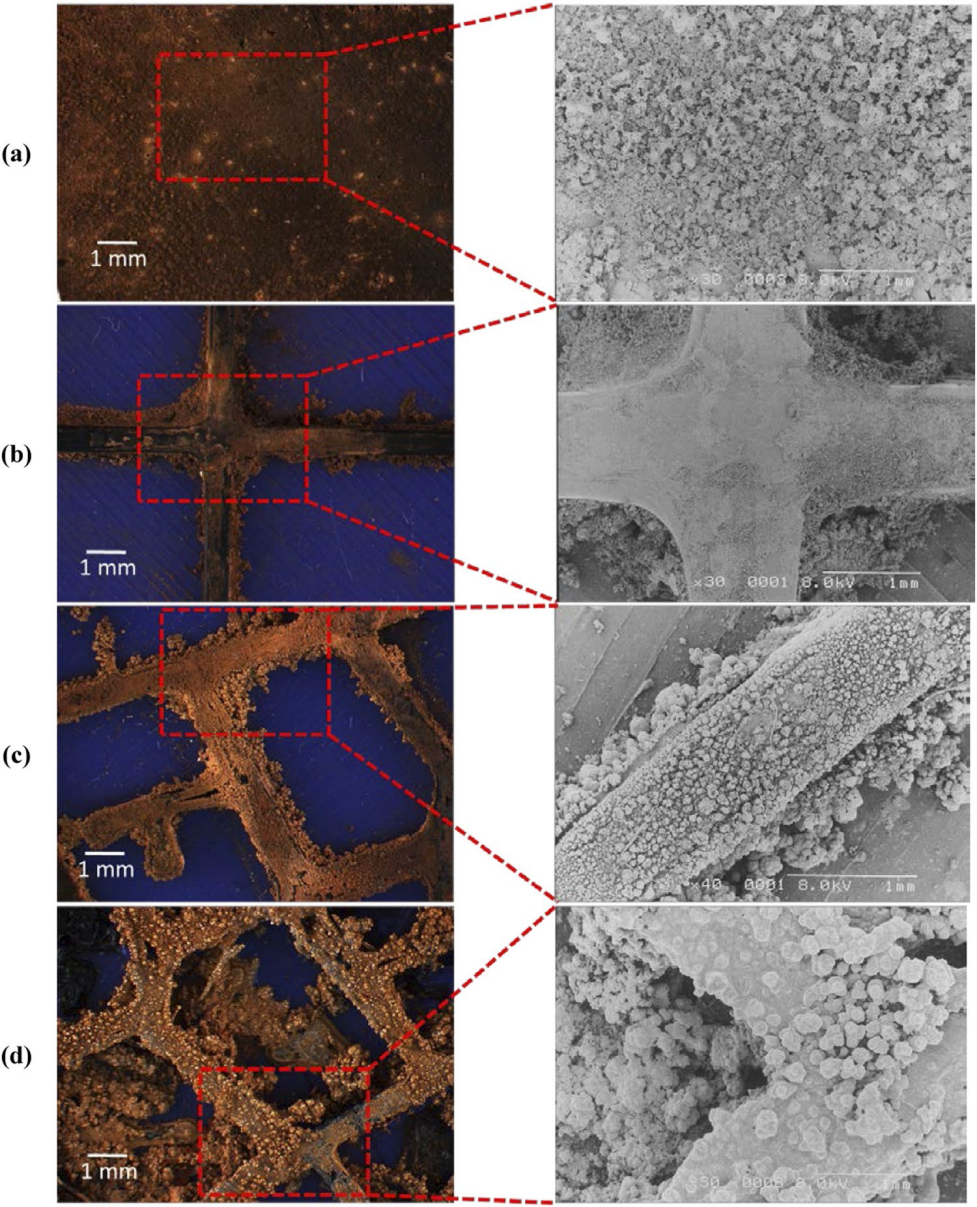
Morphology of Different Structures for CE (**a**) Simple Planar Structure (**b**) Cross Structure (**c**) 2D Fractal Structure (**d**) 3D Fractal Structure.

The morphology of the fabricated CEs with different designs including Planar, Cross, 2D Fractal and 3D Fractal are analyzed using a digital microscope (make: OMAX, Korea, Magnification 2000x) and an SEM (make: HITACHI S-2400, Japan). The digital microscope images and the SEM images of the coated CEs are shown in Fig. 6. The digital microscope images show a uniform coating of Cu/Graphite particles on the conductive PLA surface. The non-conductive ABS surface does not show signs of particle deposition. The surface morphologies as seen on the SEM images suggest that the Cu/Graphite particles project on the fractal network. The SEM images showed Cu/Graphite traces at the edges of fractal structures forming active sites during the DSSC operation.

### Electrical Characterization

**Figure 7:**
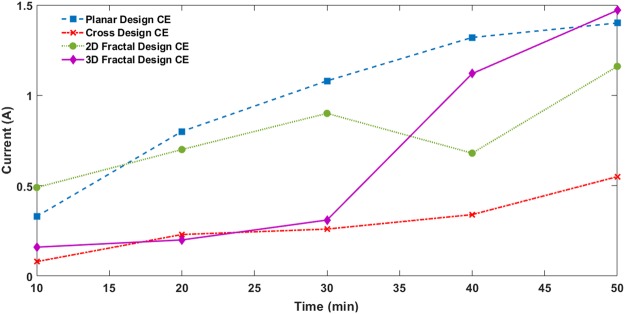
Variation in Current during Selective Electrochemical Co-deposition Process.

The mass transfer during the selective electrochemical co-deposition process increases as the current flowing through the electrolytic cell increases. The current is one of the most influencing factors affecting the thickness of the deposited layer during this deposition process. Figure 7 shows the variation in current during the deposition process for the different design of CEs. In this experiment, the voltage is maintained in the range of 6–8 volts, and the current values are measured at intervals of 10 minutes. As current value increases, the rate of Cu/Graphite deposition also increases. The figure shows the current values for the planar structure increase steadily and reaches a maximum value of 1400 mA. The cross structure shows the lowest current values among all the designs. It means that larger surface area of the conductive PLA material in planar design is capable of attracting more Cu/Graphite particles compared to cross design. Moreover, the initial deposition of Cu/Graphite layer promotes faster material deposition as the time progresses.

The 2D fractal design showed higher current flow initially compared to the cross design and lower than the planar design. It is because of the relatively larger surface area of the conductive PLA material compared to that on the cross design promoting more material deposition. Deposition process on 3D fractal structure showed small current values initially. As the time progresses, the current value rapidly increases and reaches a maximum value of 1500 mA at 50 minutes which is the highest among all the CEs. It suggests that effective deposition of Cu/Graphite is achieved in the 3D fractal structure with a significantly large number of active catalytic sites. The increased current values also mean that there would be more potential reactions to reduce the tri-iodides the CE/electrolyte interface in the DSSC assembly during its operation.

**Figure 8:**
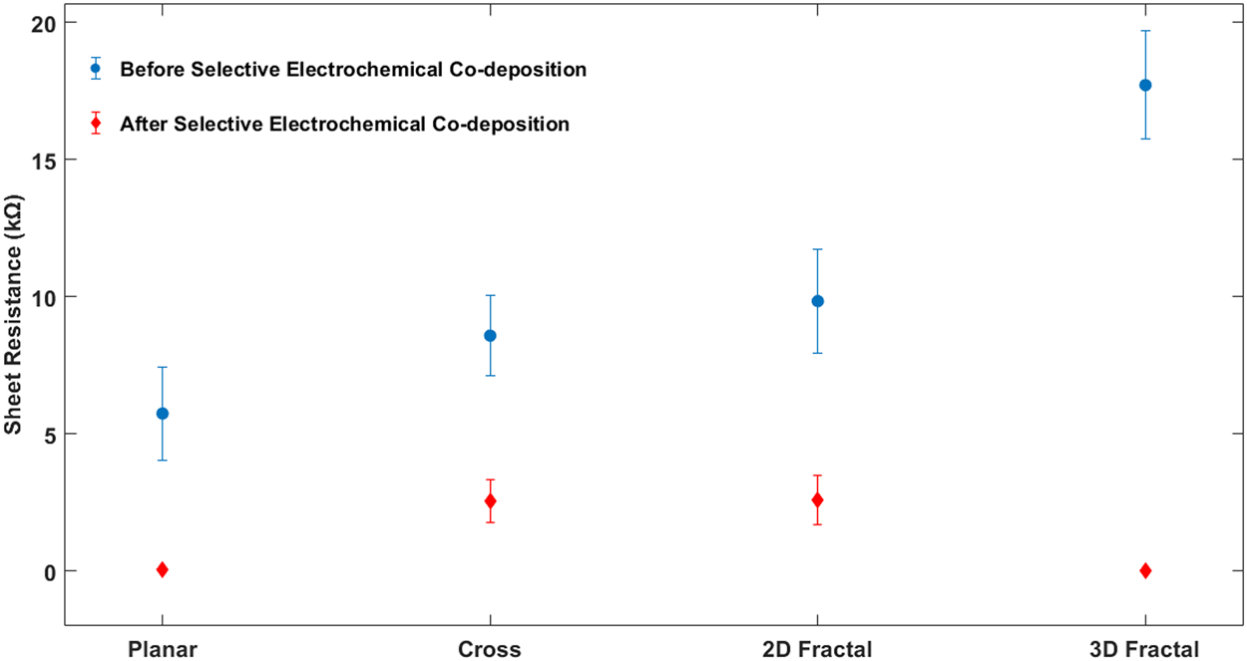
Average Sheet Resistance of CEs Before (Blue) and After (Red) the Selective Electrochemical Co-Deposition Process.

Sheet resistance is a standard measure used to characterize material deposition on thin films. It represents the measure of the resistance of deposited material on the substrate surface. In this study, the sheet resistance of each CE is measured at different points on the surface of the conductive regions before and after the selective electrochemical co-deposition process. A digital multimeter (make: Stalwart Tools, USA) is used to measure the resistance of each electrodes using a two-probe measurement technique. It is assumed that conductivity is not obtained on all data points. Therefore, the average sheet resistance and the standard deviation are calculated and is shown in Fig. 8. It is seen that after deposition sheet resistance value decreased for all the CEs. The figure shows that the 3D fractal design based CE has the lowest average sheet resistance of 0.03 kΩ compared to other CEs after deposition. It corresponds to a significant reduction (99.8%) in sheet resistance. The considerable decrease in sheet resistance for the 3D fractal design based CEs can be attributed its fractal structure helping faster and uniform ion transport. The sheet resistance reduction for other designs including planar, Cross, 2D fractal based CEs is 99%, 70.1%, and 73.6% respectively. It means that the 3D fractal design shows a more considerable reduction in sheet resistance values compared to other designs after the Cu/Graphite deposition.

**Figure 9:**
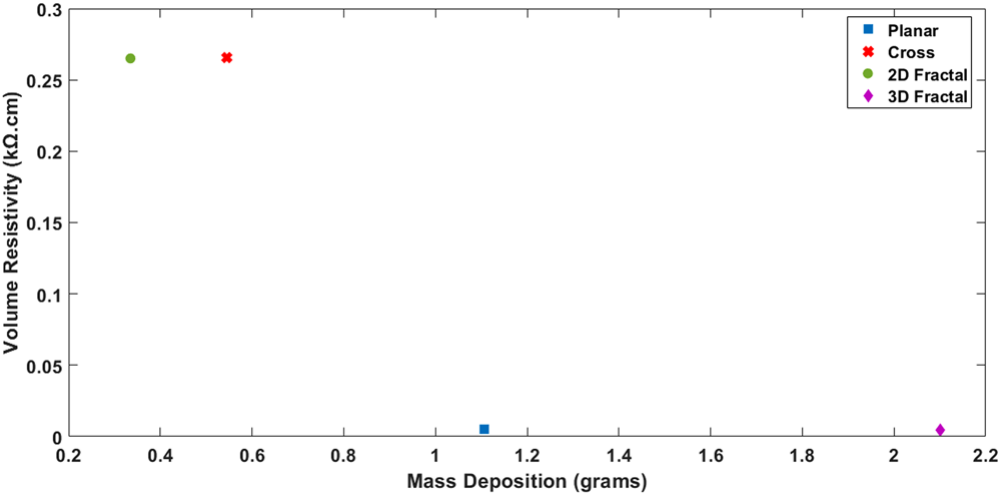
Variation in1 Volume Resistivity with respect to Mass Deposition for Different CE Designs.

The electrical resistivity (volume resistivity) of materials is a critical factor affecting its performance in electrical applications. The assumption is made that deposition on all printed electrodes is Ohmic and completely homogeneous in composition. The following equation calculates the volume resistivity.

1 $$\rho =Rt/(l/w)$$

where *ρ* is the Resistivity in Ω-cm, *R* is the average Sheet Resistance in Ω, *t* is the thickness of the deposited layer in cm, *l* and *w* are the length and width of the electrode respectively in cm. The thickness of the deposited layer is calculated by the following equation^33^.

2 $$t=\frac{{\rm{\Delta }}m}{S.{\rho }_{m}}$$

where Δ*m* is the Mass of deposition in grams, S is the Surface area of the component in cm^2^ and *ρ*_*m*_ is the Density of material in g/cm^3^. In this study, the material density is assumed to be the density of Cu since the mass of graphite is negligible. Figure 9 shows the volume resistivity variation with respect to the mass deposition of different CEs. It is seen that the resistivity decreases with an increase in the mass deposition of Cu/Graphite. CEs with planar structure and 3D fractal structure showed minimal volume resistivities of 0.0051 kΩ-cm and 0.0043 kΩ-cm respectively. The minimum resistivity of CE electrode would provide more conductivity during the photovoltaic performance. The larger mass deposition of 3D fractal design compared to planar design means that the fractal geometry is capable of providing a significantly higher number of catalytic activity sites during the DSSC operation. It is thus indicating that the CEs based on fractal designs could increase the efficiencies of the DSSCs.

## Assembly of DSSC

**Figure 10:**
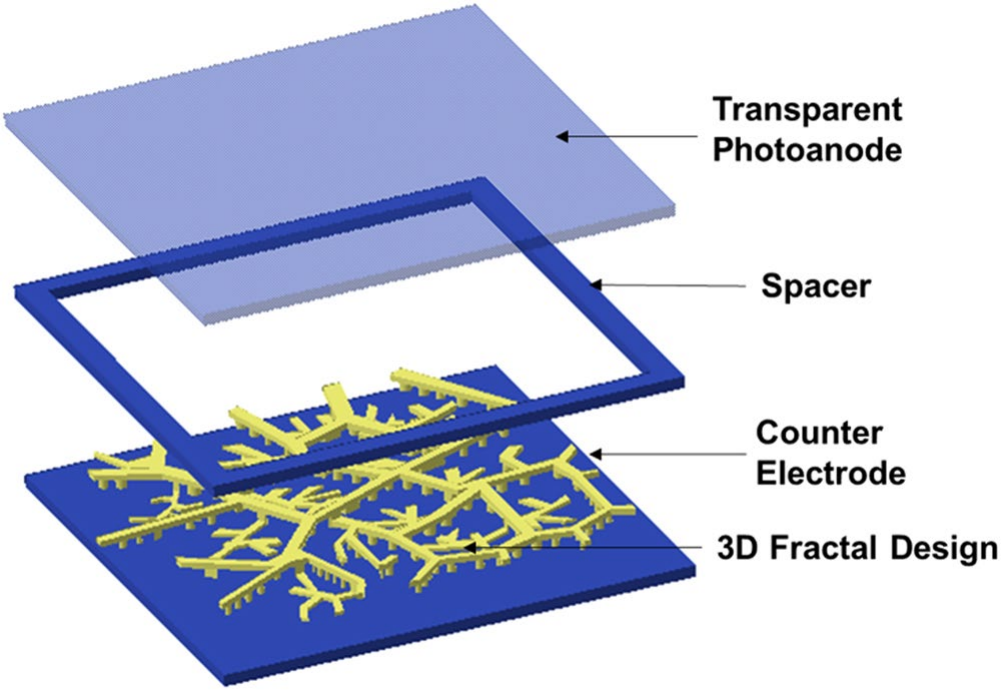
Schematic of the DSSC Assembly including the fabricated 3D Fractal Design-based CE.

The fabricated CEs are further assembled into DSSCs to understand the effectiveness of various designs. The DSSC assembly consists of the transparent photoanode, an insulating spacer layer, the fabricated CE, along with chemical ingredients including TiO_2_, sensitizing dye and the electrolyte. The schematic of the DSSC assembly used in this study is shown in Fig. 10. TiO_2_ paste is used as the semiconductor which is coated with photoexcited state of sensitizer Eosin Y Dye (92% Dye Content, certified, 25 g make: Sigma-Aldrich, USA). The electrolyte material used is Spectra Electro Gel, (60 g) in the quasi-solid-state. Table 3 shows the list of materials used for the DSSC assembly.

**Table 3:** Materials Used for DSSC Assembly.

Material	
Substrate/Photoanode	ITO-PET Transparent Sheet
Semiconductor layer	TiO_2_ Powder
Sensitizer	Eosin Y Dye
Electrolyte	Spectra Electro Gel
Sealing	Non-Conductive ABS
Substrate/CE	3D printed fractals-based Cu
Catalyst material	Graphite Particles

**Figure 11:**
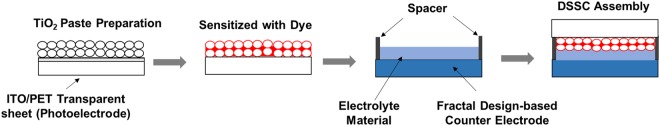
Schematic of Steps Involved in Assembly of DSSC.

**Figure 12:**
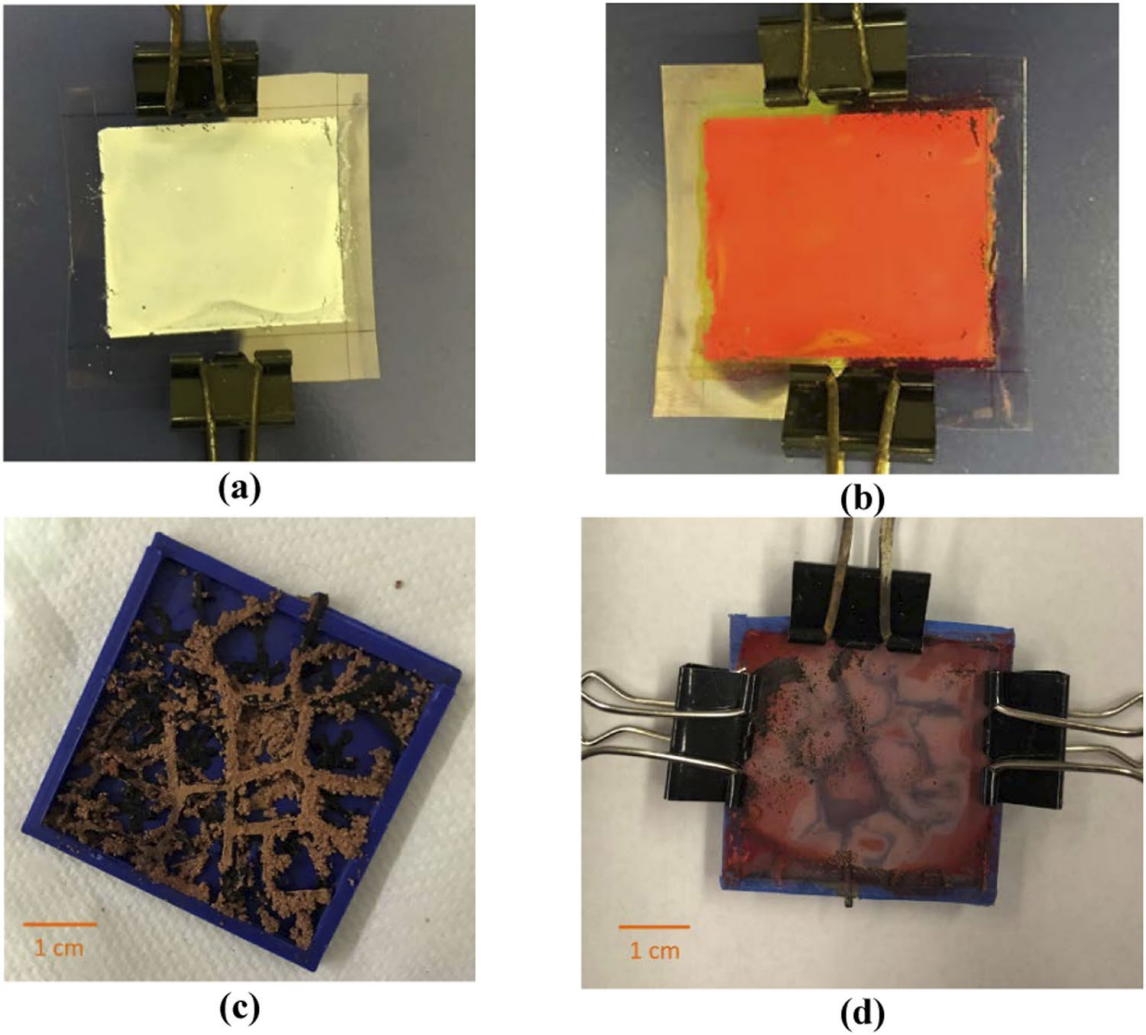
Assembly of DSSC: (**a**) Preparation of Photoanode Coated with TiO_2_ Paste (**b**) Sensitization of photoanode with Dye (**c**) Fabricated 3D Fractal-based CE (**d**) Final Assembly of DSSC with Electrolyte between Photoanode and CE.

The photoanode used for the assembly consists of conductive Indium Tin oxide coated Polyethylene Terephthalate (ITO-PET) transparent sheet (make: Sigma-Aldrich, USA, with Surface resistivity: 60 Ω/sq and Transmittance: 550 nm, >79%). A binder-free paste is prepared by mixing TiO_2_ power (2 grams) with 0.2 ml of Nitric acid. For each electrode, the prepared TiO_2_ paste is deposited on the ITO-PET transparent sheet by the Doctor Blading technique. Scotch tape is used to maintain a uniform layer of TiO_2_ paste on ITO-PET transparent sheet. It is followed by annealing of the TiO_2_ layer at low temperature (100–120 °C) using a hotplate. The prepared photoanode is then immersed into a 0.5 mL Dye solution for 2–4 hours and washed with ethanol to remove impurities of dye. A small quantity of electrolyte gel is filled on the CE surface. The dye-sensitized photoanode along with the fabricated CE is sandwiched together using a spacer. The spacer is made using non-conductive ABS material through FDM 3D printing process. Binder clips are used to encapsulate both sides of electrodes. Figure 11 shows the schematic of the detailed steps involved in the assembly of DSSC. The above steps are repeated for all the DSSCs. Figure 12 shows the assembly of DSSC including the fabricated 3D fractal based CE.

### Performance Testing of DSSCs

**Figure 13:**
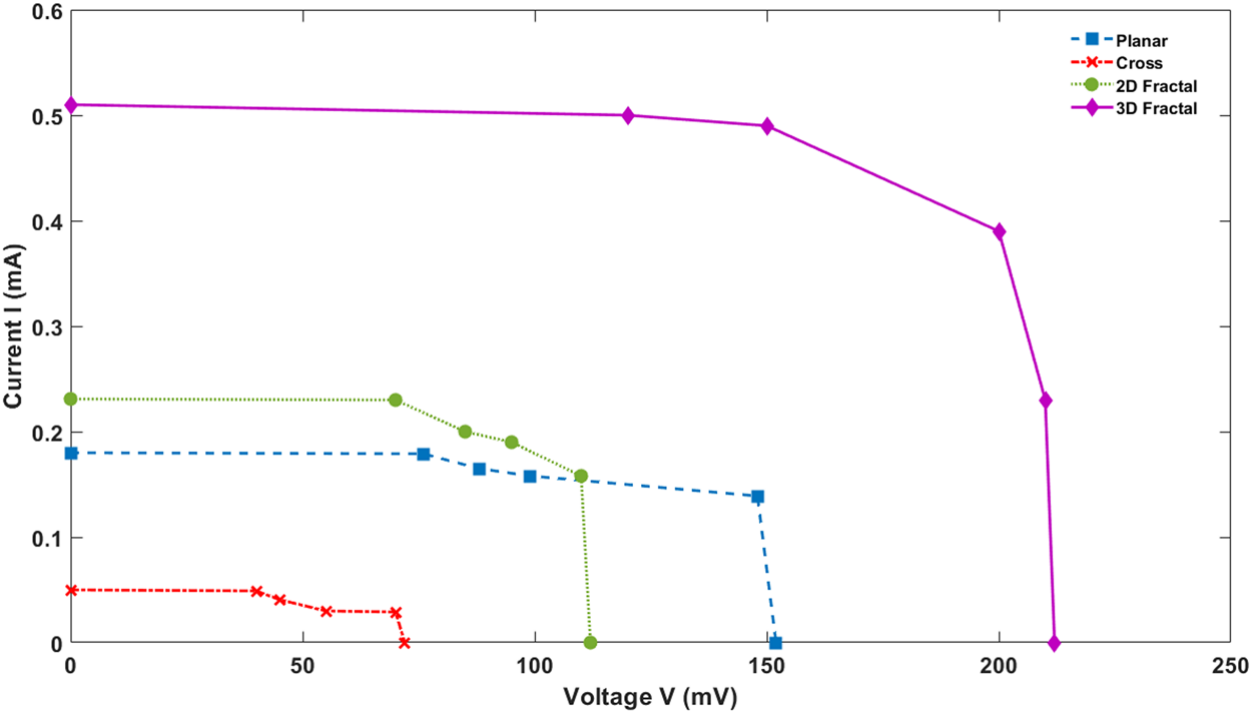
I-V curves of DSSC with different CEs under Illumination at 100 mW/cm^2^, (Air Mass Coefficient 1.5 G).

One crucial parameter to increase the efficiency of DSSC is to improve Short-circuit current (*I*_*sc*_) and equivalent flow of current through CEs. The short-circuit current correlates with the electrocatalytic activity of CEs. The Current-Voltage (I-V) measurements are carried out to evaluate the performance of DSSC fabricated using various CE designs. The I-V values are measured under standard illumination condition – Air Mass Coefficient 1.5 G at 100 mW/cm^2^. Figure 13 shows the I-V curve for the DSSC assembled using the various designs of CEs. Further, the efficiency of each DSSC is calculated using the following equation:

3 $$\eta =\frac{{I}_{sc}\times {V}_{oc}\times FF}{{P}_{in}}$$

where *I*_*sc*_ is the Short-circuit current in mA, *V*_*oc*_ is the Open-circuit voltage in mV*, FF* is the Fill factor, and *P*_*in*_ is the Power input in mW. Table 4 shows the photovoltaic parameters of DSSCs assembled with different CEs. The 3D Fractal design shows the highest short circuit current (0.5 mA) among all the DSSCs. It suggests that the 3D fractal design would provide the highest electrocatalytic activity during the DSSC operation. The corresponding efficiency of the 3D fractal design based DSSC is 4.67%. It is significantly larger than the efficiencies of DSSC made using other CE designs. It means 3D fractal design provides more active sites for electron collection and faster transportation which in turn improves electro-catalytic activity and conductivity of CE. Both planar design and 2D fractal designs showed lower short-circuit currents. There is only a minimal difference in short-circuit current between the DSSCs assembled using planar CE and 2D fractal based CE. The efficiencies of DSSCs using CEs with planar and 2D fractal based structure are 1.26% and 1.22% respectively. The DSSC with a cross design based CE has the lowest efficiency of 0.12% due to its lower short-circuit current (0.05 mA). It shows lowest catalytic activity and low conductivity on the active region. The study thus finds that the 3D type fractal based CE has a significant advantage over CEs fabricated using other designs.

**Table 4:** Photovoltaic Parameters of DSSCs Assembled with Different CEs.

	V_oc_ (mV)	I_sc_ (mA)	FF	Efficiency η %
Planar Design	150	0.18	0.75	1.26
Cross Design	60	0.05	0.67	0.12
2D Fractal Design	110	0.23	0.77	1.22
3D Fractal Design	212	0.5	0.70	4.67

## Conclusion

In this study, a novel conceptual design based on nature-inspired fractal design is proposed for counter electrodes (CEs) used in Dye-Sensitized Solar Cells (DSSCs). The fabrication on the fractal design based CEs are done using FDM additive manufacturing technique of conductive polymer materials. It is followed by a selective electrochemical co-deposition process of Copper/Graphite material. The fabricated CEs are characterized, analyzed, and performance tested. The study finds that the fractal-based design outperforms conventional designs while showing minimum electrical resistivity. The 3D fractal design provides maximum surface coverage, achieve a uniform current density, and have a minimum overall resistance for application in DSSCs. The I-V characterization showed that higher current results in increased electron collection and transportation due to fractal designs in CE. Comparison of DSSC devices fabricated using the new CEs indicated that the use of fractal-based CEs in DSSCs leads to an increased photocurrent conversion efficiency. Additionally, adopting a new additive manufacturing process makes it easier and cheaper to fabricate this type of CEs. The results of this study suggest that the fractal design based CEs used in DSSCs are a potential candidate for low-cost photovoltaic applications. Future advancement in additive manufacturing with hybrid manufacturing of liquid material and solid materials would make it suitable for improvement in more massive production for DSSCs.

## Data Availability

The datasets generated during the current study are available from the corresponding author on reasonable request.
